# Microbial Degradation of Epoxy

**DOI:** 10.3390/ma11112123

**Published:** 2018-10-29

**Authors:** Noam Eliaz, Eliora Z. Ron, Michael Gozin, Sara Younger, Dvora Biran, Noam Tal

**Affiliations:** 1 Department of Materials Science and Engineering, Faculty of Engineering, Tel-Aviv University, Ramat Aviv, Tel Aviv 6997801, Israel; sara.sofer@gmail.com; 2 School of Molecular Cell Biology and Biotechnology, Faculty of Life Sciences, Tel-Aviv University, Ramat Aviv, Tel Aviv 6997801, Israel; eliora@post.tau.ac.il (E.Z.R.); birand@post.tau.ac.il (D.B.); 3 School of Chemistry, Faculty of Exact Sciences, Tel-Aviv University, Ramat Aviv, Tel Aviv 6997801, Israel; cogozin@tauex.tau.ac.il; 4 Mass Spectrometry Laboratory, School of Chemistry, Faculty of Exact Sciences, Tel-Aviv University, Ramat Aviv, Tel Aviv 6997801, Israel; noamtal@tauex.tau.ac.il

**Keywords:** epoxy, epoxy resin, biodegradation, microbial degradation

## Abstract

Epoxy resins have a wide range of applications, including in corrosion protection of metals, electronics, structural adhesives, and composites. The consumption of epoxy resins is predicted to keep growing in the coming years. Unfortunately, thermoset resins cannot be recycled, and are typically not biodegradable. Hence, they pose environmental pollution risk. Here, we report degradation of epoxy resin by two bacteria that are capable of using epoxy resin as a sole carbon source. These bacteria were isolated from soil samples collected from areas around an epoxy and polyurethanes manufacturing plant. Using an array of molecular, biochemical, analytical, and microscopic techniques, they were identified as *Rhodococcus rhodochrous* and *Ochrobactrum anthropi*. As epoxy was the only carbon source available for these bacteria, their measured growth rate reflected their ability to degrade epoxy resin. Bacterial growth took place only when the two bacteria were grown together, indicating a synergistic effect. The surface morphology of the epoxy droplets changed significantly due to the biodegradation process. The metabolic pathway of epoxy by these two microbes was investigated by liquid chromatography mass spectrometry. Bisphenol A, 3,3′-((propane-2,2-diylbis(4,1-phenylene))bis(oxy))bis(propane-1,2-diol) and some other constituents were identified as being consumed by the bacteria.

## 1. Introduction

*Epoxy resins* contain epoxide groups, and may be reacted (cross-linked) with a wide range of co-reactants (also known as hardeners), such as amines and phenols. This cross-linking reaction is commonly referred to as curing, and it forms thermosetting polymers. Epoxies are known for their excellent adhesion, chemical and heat resistance, good-to-excellent mechanical properties, and very good electrical insulating properties. They have a wide range of applications, including paints and coatings (e.g., in corrosion protection of metals), electronics and electrical insulators, structural adhesives, and fiber-reinforced composites (e.g., with carbon fiber and fiberglass reinforcements). The global demand for epoxy resins was estimated at ~21.5 billion USD in 2016, and is forecasted to increase to 37.3 billion USD by 2025 [1].

*Biodegradation* is one of various polymer degradation routes. It is the process by which organic substances are broken down by microorganisms, such as bacteria, fungi, and algae. These microorganisms can degrade the polymers aerobically—producing carbon dioxide and water, or anaerobically—producing carbon dioxide, water, and methane [2]. Degradation of synthetic polymers (plastics) can take place by microbial utilization of their carbon backbone as a carbon source. 

Unlike thermoplastics, *thermoset resins* cannot be recycled. Furthermore, they are typically not biodegradable, and can only be burned under strict precautions due to the release of toxic substances. Consequently, environmental pollution by their use and waste has been recognized as a severe problem, which must be globally addressed. A recently published paper describes a study of the fungal and bacterial microflora isolated from an epoxy art object, which can grow on the surface of the object [3]. That study raises the possibility that microorganisms that are capable of degrading epoxy exist. While it is well known that epoxy is often used to protect from corrosion [4], we could find only one reference to microbiologically influenced corrosion (MIC) of the protecting plastic itself. Wang et al. [5] explored the degradation of epoxy resin varnish coating in both sterile seawater and seawater inoculated with *Pseudomonas* putida, which was isolated from natural seawater with 2216E medium. The bacterium significantly decreased the corrosion resistance of the coating. The bacterium formed a mature biofilm on the coating and promoted extensive underfilm corrosion of the steel substrate. The authors attributed the degradation of the epoxy in the presence of the bacterium to oxidation of hydroxyl to the intermediate carbonyl (aldehyde).

Here, we describe our study aimed to isolate from the surroundings microorganisms that can degrade thermoset epoxy resins in an economically viable process under mild conditions, characterize these microorganisms, demonstrate the biodegradation process, and try understanding the metabolism of epoxy by these microorganisms. This study could open the door also to biodegradation of the cured end-products of epoxy resins, which might be a more challenging task. Such a microbial degradation process is a promising eco-friendly strategy, which represents a great opportunity to manage waste plastic materials with no adverse impacts. The discovery of such plastic-degrading microorganisms has great biotechnological potential, may become part of a larger screening program, and could aid natural bioremediation processes, favoring the natural cleaning of natural ecosystems.

## 2. Materials and Methods

### 2.1. Materials

Two types of epoxy resin were used: (1) Araldite^®^ LY 5052 (Huntsman Advanced Materials Americas LLC, The Woodlands, TX, USA). This low-viscosity resin is a blend of 1,4-butanediol diglycidyl ether (C_10_H_18_O_4_) and epoxy phenol novolac resin (C_35_H_32_O_4_), which contains bisphenol A. The content of these two constituents is 55–68 and 38–42 wt.%, respectively. The weight per epoxide (per ASTM D1652) of this resin is 148.33 g/eq. The number of epoxide groups in the phenol novolak resin is four, and its molecular weight is 345 g/mol. The functionality of 1,4-butanediol diglycidyl is two, and its molecular weight is 202.3 g/mol. The main applications of the Araldite^®^ 5052 system are in aerospace and industrial composites, and in tooling for aircraft repair. (2) EPON™ resin 815C (Momentive Specialty Chemicals, Inc., Columbus, OH, USA). This low-viscosity resin is a blend of 4,4-Isopropylidenediphenol-Epichlorohydrin copolymer (containing bisphenol A) and 2-(butoxymethyl)oxirane. The latter is also referred to as n-butyl glycidylether. The weight per epoxide of this resin is 180–195 g/eq, and its number average molecular weight is ≤700.

### 2.2. Soil Samples

Soil samples were collected from different regions outside the manufacturing factory of Polymer-G (Kibbutz Gvulot, southern Israel, http://www.polymer-g.com), including from the front door. This factory makes epoxy, polyurethanes, and silicones.

### 2.3. Enrichment and Growth of Bacteria

1 g of soil sample was added to 10 ml of Davis minimal medium (broth) [6]. The composition of the Davis medium: 7 g/L K_2_HPO_4_, 3 g/L KH_2_PO_4_, 1 g/L (NH_4_)_2_SO_4_, 0.5 g/L Na_3_-Cit·3H_2_O, 0.1 g/L MgSO_4_·7H_2_O, and its pH 7.0 ± 0.2 at 25 °C. Minimal media are those that contain essential minerals and a carbon source. We added 1 wt.% of epoxy resin as the carbon source. The samples were kept under aerobic conditions at 30 °C while shaking at 150 rpm for one week. Some cultures were grown on Difco™ LB Broth, Lennox. Exponentially growing cultures were diluted to an optical density at 600 nm (OD_600nm_) of 0.03. In order to enrich the bacteria growing on the epoxy media, 100 μL of a one-week old culture was added to a fresh 10 mL epoxy resin growth medium. The soil was allowed to settle for one hour before a sample was taken from the top of the suspension for a second enrichment in Davis medium. Overall, five enrichment cycles were run at one-week intervals, and the turbidity was measured at OD_600nm_ after each cycle.

### 2.4. Turbidity (OD_600nm_) Measurements

Turbidity measurements were made at 10 min steps, using a BioTek^®^ Eon™ microplate monochromator-based spectrophotometer (BioTek Instruments, Inc., Winooski, VT, USA). The underlying principle is that most of the light scattered by the bacteria no longer reaches the photoelectric cell, so that the electric signal is weaker than for a bacteria-free cuvette. The choice of 600 nm wavelength is due to the fact that many of the components that comprise the cell culture media remain transparent at this wavelength, so that optical density is proportional to the density of cells in the light path. OD_600nm_ is preferable to ultraviolet (UV) spectroscopy when measuring the growth over time of a cell population because, at this wavelength, the cells will not be killed as they would under too much UV light. UV light has also been shown to cause small to medium sized mutations in bacteria, potentially altering or destroying genes of interest.

### 2.5. Identification of Bacteria

Genomic identification of specific bacteria was based on sequencing of the gene coding for 16S RNA [7,8,9,10]. Genomic DNA was isolated using Wizard^®^ Genomic DNA Purification Kit (Promega Corp., Madison, WI, USA). DNA fragments were separated via 1% agarose gel electrophoresis. The gels were prepared by dissolving the agarose in a Tris-acetate-EDTA (TAE) buffer. Polymerase chain reaction (PCR) was used to amplify the DNA segment that can provide bacterial identification. The primers used for amplifying the conserved regions within 16S rRNA genes were: forward primer AGAGTTTGATCCTGGCTCAG, reverse primer GGTTACCTTGTTACGACTT. For sequencing of an amplified material, a colony was mixed with 1 μL of each primer, 5 μL Taq polymerase buffer, 5 μL deoxynucleotide triphosphates (dNTPs), 0.5 μL TaKaRa Ex Taq™ DNA polymerase enzyme, and water to 50 μL. The amplification conditions were as follows: 30 cycles at 95 °C for 5 min, 94 °C for 1 min, 50 °C for 1 min, 72 °C for 1 min, a final extension at 72 °C for 5 min, and cooling to 10 °C for 1 min [11]. The DNA was detected using UV light, and the size of the DNA was determined using a standard 1 kb DNA Ladder. The DNAs were cleaned using ExoSAP-IT™ reagent (Affymetrix, Inc., Cleveland, OH, USA), and were subsequently sequenced at the sequencing unit of Tel-Aviv University. Bacterial 16S-rRNA sequences were obtained from GenBank^®^ NIH genetic sequence database. Partial 16S rRNA sequences were compared with sequences deposited in the database, using the standard nucleotide-nucleotide Basic Local Alignment Search Tool (BLAST^®^) code (http://www.ncbi.nlm.nih.gov/blast/). To avoid errors due to the alignment of DNA sequences of varying lengths, only the first 1500 nucleotides in each sequence were used for the alignment.

VITEK^®^ 2 (bioMérieux, Inc., Durham, NC, USA) [9,12] is an automated microbiology system utilizing growth-based technology for the identification and antimicrobial susceptibility testing (AST) of microorganisms. It identifies the organism and its antibiotic sensitivity by detecting color changes or turbidity in special plastic cards inoculated with the organism. The optical system combines multichannel fluorimeter and photometer readings to record fluorescence, turbidity, and colorimetric signals. Following Gram staining, cultures were grown at 35 °C on either sheep blood or MacConkey agar plates for 24 h. The VITEK^®^ 2 system with anaerobic and corynebacteria identification card (ANC) was then used for the automated identification of the bacteria according to the manufacturer’s procedure.

In matrix-assisted laser desorption ionization–time-of-flight mass spectrometry (MALDI-TOF MS) [13,14,15], a sample slide is prepared and is introduced into a high-vacuum measurement chamber. The sample is ionized with a precise laser burst. The ionized compounds are accelerated in a flight tube using an electric charge, and their time-of-flight is recorded; lighter proteins travel faster, heavier proteins travel slower. At the end of their travel, the proteins are detected with a sensor, which creates a spectrum representing the protein makeup of each sample. By comparing the spectrum from a particular sample against a large database of spectra from precisely characterized bacteria and fungi, identifications can be made at the species, genus, and family level. MALDI-TOF MS was conducted using a VITEK^®^ MS system (bioMérieux, Inc., Durham, NC, USA), following the manufacturer’s procedure. Briefly, a portion of a fresh colony was smeared on a FlexiMass™ disposable target plate, and was then immediately covered with 1 µL of ready-to-use α-cyano-4-hydroxycinnamic acid (CHCA) matrix solution. After drying at room temperature, the target plate was loaded into the Axima Assurance mass spectrometer. Spectra were generated and compared to a database that contains 193 clinically relevant microorganisms.

The shape and size of the bacteria were characterized using a Nikon Eclipse TE2000-U (Nikon Instruments, Inc., Melville, NY, USA) inverted light microscope equipped with bright field, phase contrast, and epi-fluorescence cubes. Colonies were transferred from LB growing plates to a glass slide with double-distilled water (DDW). A coverslip was placed over the root or entire seedling, to ensure minimal trapped air bubbles, and the excess liquid was drained from the slide.

### 2.6. Demonstration of the Biodegradation of Epoxy Resin by Bacteria

The surface morphology of epoxy droplets, in the absence as well as in the presence of bacteria, was characterized by FEI Quanta 200 field-emission gun (FEG) environmental scanning electron microscope (ESEM™), operated in the wet mode with a gaseous secondary electrons detector (GSED). The ESEM vacuum system was pumped down to 5.3 Torr, which provided relative humidity of 100% for a sample temperature of 2 °C, as described elsewhere [16]. The clear difference between the surface morphology of droplets in the reference (bacteria-free) versus bacteria-containing sample under the same imaging conditions, as well as the match in shape and size of the two bacteria analyzed to those reported in several previous references, and to our own light microscope data, helped us confirming that the droplet surface features were not artifacts.

The metabolic pathways of the epoxy resins by bacteria were studied by the liquid chromatography–mass spectrometry (LC-MS) technique. LC-MS combines the resolving power of LC with the detection specificity of MS. LC separates a sample to its components, and then introduces them to the mass spectrometer for further analysis. The LC-MS data can be used to provide information on the molecular weight, structure, identity and quantity of specific sample components. In this study, we used a SYNAPT^®^ G1 high-resolution Q-TOF LC-MS mass spectrometer equipped with an ACQUITY™ UPLC^™^ system and a tunable UV (TUV) detector, which is capable of UV-vis detection and electrospray ionization in the same run (Waters Corporation, Milford, MA, USA). The stationary phase consisted of a C18 (1.7 µm, 2.1 × 100 mm) column (Waters Corp., Milford, MA, USA), and the mobile phase compositions were: (A) 95% H_2_O + 5% acetonitrile + 0.1% formic acid; (B) acetonitrile + 0.1% (*v*/*v*) formic acid. The elution gradient consisted of a linear increase to 100% B in 12 min and hold for 2 min, then return to the starting conditions for additional 1 min. 10 µL samples of analyzed materials were injected, and the flow rate was 0.3 mL/min. The temperature in the sample chamber was preset to 10 °C, and remained stable (±1 °C) throughout the measurement. The UV detector was set to 254 nm. The mass spectrometer was operated both in the negative and in the positive ion modes. Data interpretation was done for both ion modes, using MassLynx™ software (v4.1, Waters Corp., Milford, MA, USA).

## 3. Results and Discussion

### 3.1. Enrichment for Bacteria Capable of Degrading Epoxy Resin

**Figure 1:**
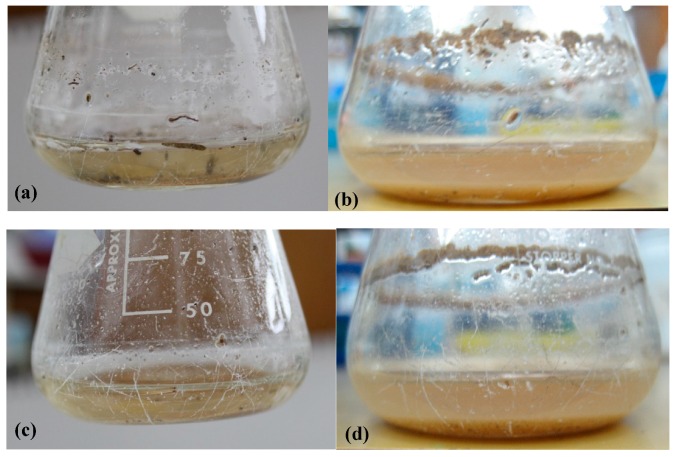
Flasks containing Davis minimal medium, soil samples, and epoxy resins before (**a**,**c**) and after (**b**,**d**) one-week incubation. (**a**,**b**) LY 5052 epoxy resin; (**c**,**d**) 815C epoxy resin.

For the first screening stage, we incubated two different epoxy resins—Araldite^®^ LY 5052 and EPON™ resin 815C—with Davis minimal medium and soil samples. After a week of incubation at 30 °C, the cultures were diluted into fresh medium, and incubations were continued. At one-week intervals, the turbidity (OD_600nm_) of suspensions was measured, as an indicator to the growth of microorganisms. It should be noted that the oily-epoxy resin appeared as large droplets, without forming a dispersion. Since the droplets were large, they did not affect the OD value. Pictures of the flasks before and after the first week of incubation are shown in Figure 1. It is evident from this figure that the growth medium became more turbid after one-week incubation, indicating that the soil indeed contained at least one bacterium that grows by consuming carbon from the epoxy resin. In contrast, the samples which were not inoculated with soil (control samples) remained clear. Between enrichment cycles 2 and 5, the OD_600nm_ values surprisingly decreased, just opposite to the expected trend. There are two possible explanations to this phenomenon. First, it is possible that some nutrients derived from the soil ran out. The second explanation is the effect of the diversity of microorganisms in the soil. Initially, there was a large variety of microorganisms that were either specific or nonspecific to epoxy as a carbon source. As the process continued, the diversity decreased, and only microorganisms that are specific to epoxy remained.

**Figure 2:**
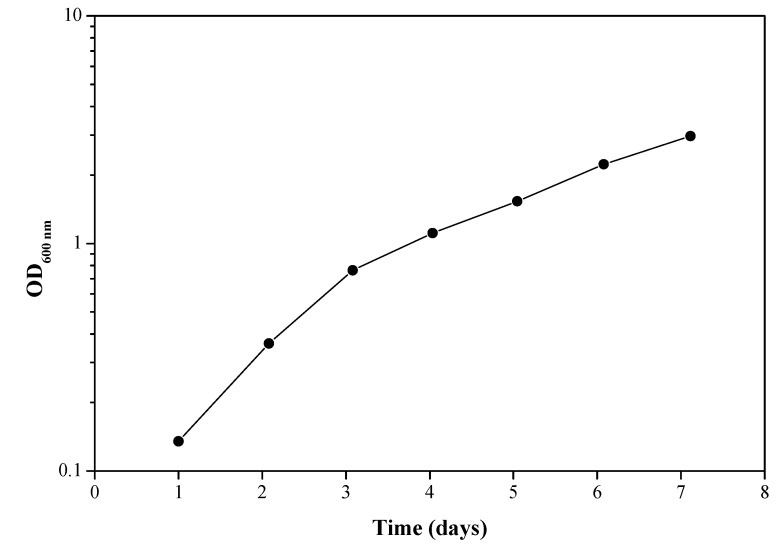
Bacterial growth over time in a culture sample after the 3rd enrichment. Carbon source: LY 5052 epoxy resin. Each experiment was repeated at least five times; a typical result is shown here.

A culture sample after the third enrichment cycle was used to determine the bacterial growth rate. When the epoxy resin is the only carbon source available for the bacteria, their growth rate can be related to the extent or rate of biodegradation of the epoxy resin. The results presented in Figure 2 show bacterial growth over time in Davis minimal medium when the source of carbon is LY 5052. The slow bacterial growth rate, with a doubling time of about 48 h, is probably due to the significantly low solubility of the epoxy resin in water. It should be noted that every experiment contained a control sample, which was not inoculated with bacteria. These control cultures were always clear, with no turbidity, and sterile.

**Figure 3:**
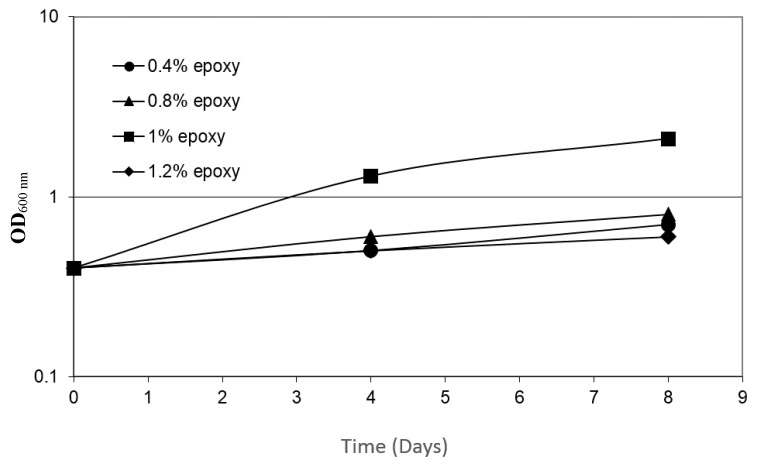
The dependence of bacterial growth rate on the concentration of LY 5052 epoxy resin. Each experiment was repeated at least five times; a typical result is shown here.

In order to determine the effect of the concentration of epoxy resin LY 5052 on bacterial growth, cultures were grown for 8 days, using media with progressively increased epoxy resin concentrations of 0.4 wt.%, 0.8 wt.%, 1 wt.%, and 1.2 wt.%. The maximal growth rate was evident at 1 wt.% (Figure 3); lower concentrations were insufficient. On the other hand, at epoxy resin concentrations above 1.2 wt.%, the media became toxic, and the bacterial growth rates declined to values significantly below the growth rates that were observed at 0.4 wt.% epoxy. The rates of biodegradation have been reported to greatly depend on the composition, state, and concentration of the carbon source, as well as on the temperature, oxygen availability, and nutrient concentrations [17]. If the concentration of the nutrient is too low, enzymes used for metabolism might not be induced; on the other hand, if it is too high, the compound might be toxic [18].

### 3.2. Characterization of the Bacteria

In order to isolate and characterize the bacteria capable of degrading the epoxy, a sample of the bacteria (after the second enrichment cycle) was grown on rich medium agar plates (LB). The plates were examined after 24 h of incubation, and it was possible to observe a clear growth of three different types of colonies, which could be distinguished based on their color: orange, yellow, or white. Because we found that the yellow-colored bacteria could be grown on different growth media only in the presence of the bacteria producing orange or white color, it was decided not to use further yellow-colored bacteria in this study.

**Figure 4:**
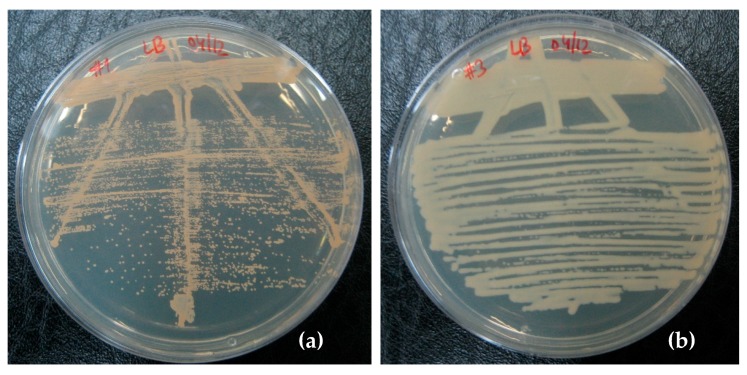
Bacteria isolated on LB agar plates. (**a**) *Rhodococcus rhodochrous*, and (**b**) *Ochrobactrum anthropi* bacteria.

**Figure 5:**
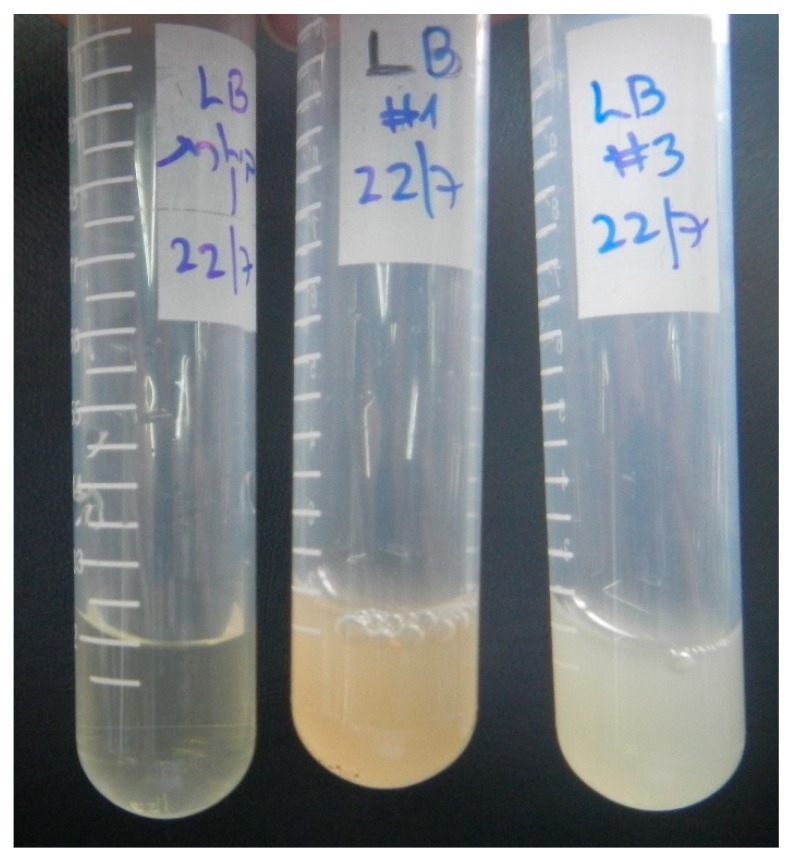
LB overnight starters. **Left**: control (no bacteria), **Center**: *Rhodococcus rhodochrous*, **Right**: *Ochrobactrum anthropi*.

The typical colonies of the other two bacteria are shown in Figure 4. The bacteria with orange- and white-colored colonies could be grown in liquid medium and retained their pigments under these growth conditions as well (Figure 5).

Identification of the bacteria was done by 16S ribosomal RNA (rRNA) gene sequencing [7,8,9]. Sequencing data for the bacteria producing white colonies showed a 97% homology with *Ochrobactrum anthropi* (chromosomes 1 and 2), ATCC^®^ 49188™, accession NC 009668.1. Sequencing data for the bacteria giving orange colonies showed 96% homology with *Bacillus* sp. 1NLA3E bacterium (accession NC 021171.1). The sequence analyses were made using the standard nucleotide-nucleotide BLAST^®^ search tool.

As our isolations started from a soil sample, and because culturable bacteria represent only a very small fraction of the bacterial population in soil, the question arises whether the two isolated bacteria are the ones who are degrading the epoxy. Our results indicate that the bacteria we cultured are indeed the ones involved in the degradation, as we could not detect additional bacteria by 16S RNA sequencing. 

**Figure 6:**
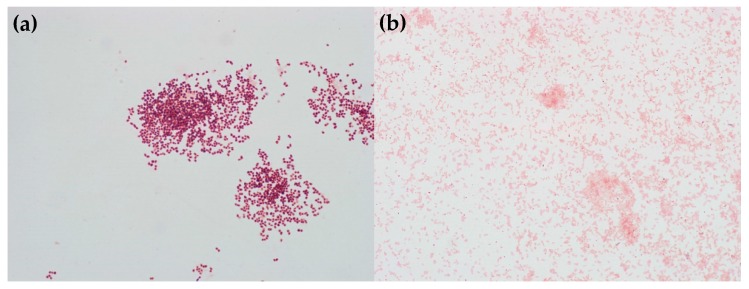
Gram staining of (**a**) Rhodococcus rhodochrous, and (**b**) Ochrobactrum anthropi.

**Figure 7:**
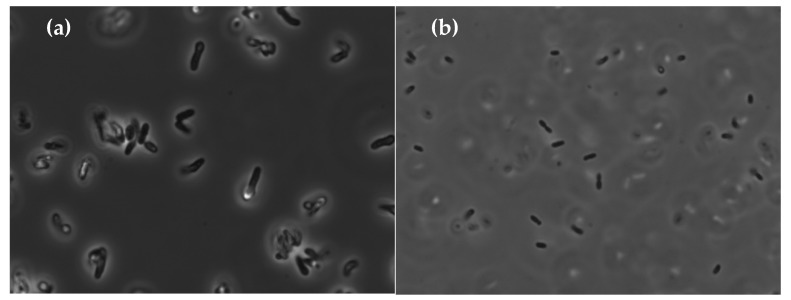
Epifluorescent microscope images revealing the rod-shape of (**a**) *Rhodococcus rhodochrous*, and (**b**) *Ochrobactrum anthropi* bacteria.

The identification of *O. anthropic* was supported by additional tests: it is Gram negative [19] (Figure 6b) and rod-shaped [20] in the epifluorescent microscope (Figure 7b). Further support was obtained by VITEK^®^ 2 [9,12]. The tested strain had the following positive substances: ellman, glycine arylamidase, l-lactate alkalinisation, citrate, d-tagatose, urease, tyrosine arylamidase, l-proline arylamidase (ProA), l-pyrrolydonyl-arylamidase (PyrA), and adonitol. These results are in line with the report of Laffineur et al. [21] that l-pyrrolydonyl-arylamidase (PyrA) and alkaline phosphatase (PHO) can be useful in identification of Gram-negative bacteria; 100% of the *Ochrobactrum anthropi* strains were found to be positive for PyrA and negative for PHO assays. 

In contrast, the bacteria giving the orange color, tentatively identified as *Bacillus* sp., were Gram-positive large rods (Figure 6a and Figure 7a). They could not be identified by VITEK^®^ 2 due to its limited database.

The last analytical technique used to identify the two bacteria was MALDI-TOF MS [13]. Using this technique, the bacteria that produced orange-colored colonies were identified as *Rhodococcus rhodochrous*, while the bacteria producing white-color colonies were identified as *Ochrobactrum anthropi*.

*Ochrobactrum anthropi* is one of nine species of the genus *Ochrobactrum*. It is an aerobic, Gram-negative, rod-shape alphaproteobacterium [22,23,24]. After growth on nutrient agar, the colonies are about 1 μm in diameter, non-pigmented, low convex, smooth, and shiny. It is chemo-organotrophic, using various amino acids, organic acids, and carbohydrates as its carbon sources. It also reduces both nitrate and nitrite by assimilation. Although cases of *O. anthropi* being pathogenic are rare, there are several documented cases showing that it is possible for individuals with an underlying medical condition, in particular those with an indwelling medical device, such as catheters and drainage tubes [25,26,27]. This is likely due to its ability to adhere to various synthetic materials. Chang et al. have shown that *Ochrobactrum sp.* bacterial strains, which were isolated from soil samples, were capable of aerobically degrading nonylphenol (NP), using it as a carbon source [28].

*Rhodococcus* is a bacterial genus that can be isolated from a range of sources, including soil, marine sediments, rocks, and groundwater [29,30,31]. It is a Gram-positive, aerobic, non-motile, mycolate-containing, nocardioform actinomycetes [29]. *Rhodococcus* exhibits a large metabolic diversity, and as a result can degrade a variety of pollutants, which may afford both environmental and industrial purposes, in transformation and degradation chemistries. *Rhodococci* can catabolize short- and long-chain alkanes, aromatic compounds (halogenated and nitro-substituted), as well as heterocyclic and polycyclic aromatic compounds. Surprising is the extent to which these bacteria extend their biodegradative abilities to compounds that could be described as more difficult with respect to recalcitrance and potential microbial toxicity [32]. Much of the biotechnological interest in *Rhodococcus* stems from the diverse range of reactions, sometimes novel, which their enzymes can catalyze. Their hydrophobic, mycolate-containing cell envelope structure also seems to be relevant to many potential applications (e.g., bioremediation, biodegradation, biotransformations, and biosurfactants) [30,33]. Difficulty in the classification of *Rhodococcus* has been attributed both to its genomics and morphology [34,35]. It suffers from genomic instability that has been related to the *Rhodococci* DNA undergoing recombination easily and at high frequency with heterologous sequences. On the other hand, the morphology of the genus also makes classification complicated. *Rhodococcus* that are in exponential phase of growth exhibit filament or short-rod shape, while those in the stationary phase tend to be in cocci [35]. *Rhodococcus* also has recalcitrant cell walls, which makes nucleic acids extraction difficult [34].

### 3.3. Growth on Epoxy Requires the Presence of the Two Bacteria

**Figure 8:**
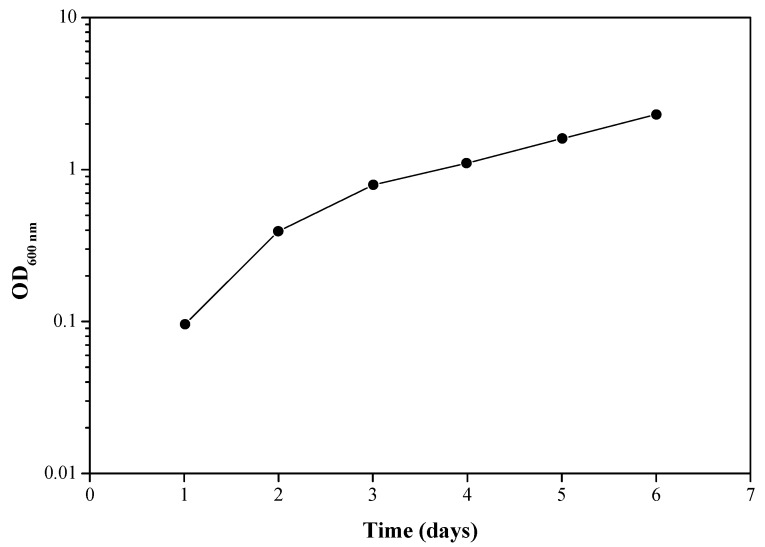
Growth of a mixture of *Ochrobactrum anthropi* and *Rhodococcus rhodochrous* on Davis epoxy LY 5052 growth medium. Each experiment was repeated at least five times; a typical result is shown here.

Once the two bacteria were successfully isolated and identified unambiguously, the growth rate of each individual bacterium was measured on various growth media. Each of the two bacteria could easily be grown on a variety of media, including a minimal medium with sodium acetate as a carbon source. In contrast, none of these bacteria could grow on a minimal medium containing epoxy resin 815C or LY 5052 as a sole carbon source. Growth on epoxy resin required the presence of the two bacteria together (Figure 8).

The nature of this obligatory synergy is not yet understood. The obligatory synergy could be due to a situation where each of the bacteria produces only part of the required enzymes for breaking down the polymer. A more complex situation may be that one of them is producing enzymes that can assist the other one via a regulatory process, such as quorum sensing (QS). QS allows bacteria to communicate via exchange of extracellular signaling molecules, called autoinducers (AIs), and act as multicellular organism. At certain concentration levels in bacterial surroundings, these AIs bind to specific receptors and initiate the expression of target genes required for biofilm formation. Acyl homoserine lactones (AHLs) are among the most extensively studied AIs produced by Gram-negative bacteria, whereas autoinducing peptides (AIPs) are the signals found in Gram-positive bacteria [36,37,38]. Most AIs are specific to a particular species of bacteria, but AI–2, which is produced by a large number of bacterial species, has been suggested to serve as a ‘universal’ signal for interspecies communication [36,38,39]. We could not find any report of QS between *Ochrobactrum anthropi* and *Rhodococcus rhodochrous*. This would worth further research.

### 3.4. The Bacteria Change the Surface Properties of the Epoxy Resin

**Figure 9:**
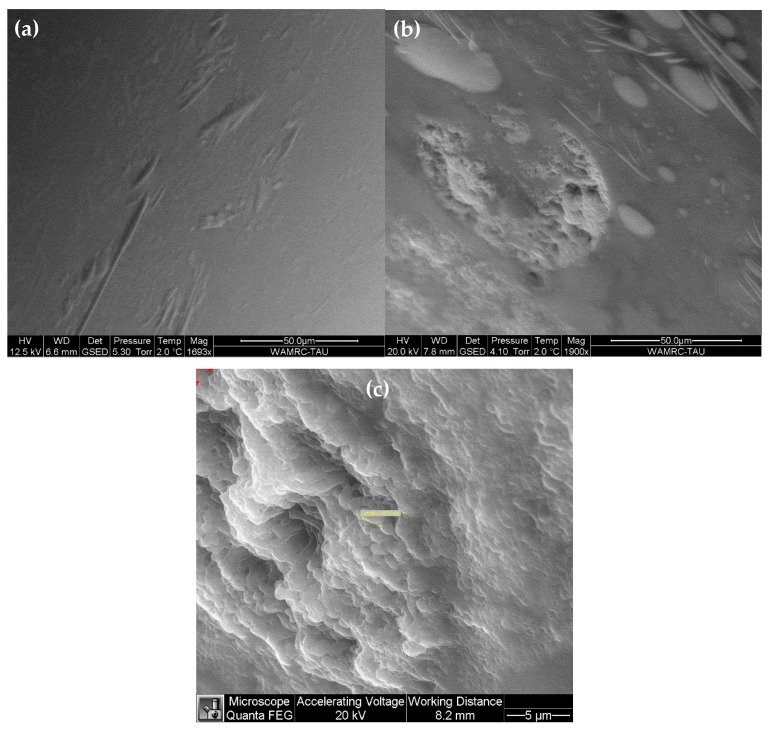
ESEM images revealing the surface morphology of: (**a**) epoxy LY 5052 droplets in the absence of bacteria; (**b**,**c**) epoxy LY 5052 droplets in the presence of bacteria, at two different magnifications.

**Figure 10:**
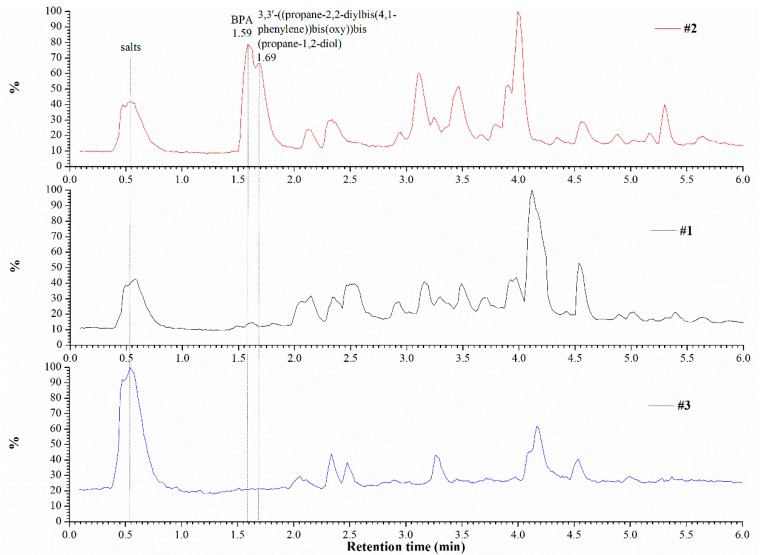
Chromatograms of the three sample compositions in the positive mode of the mass spectrometer. #1—Davis epoxy resin LY 5052 with bacteria, #2—Davis epoxy resin LY 5052 without bacteria, #3—Davis and bacteria without epoxy resin.

Finally, the biodegradation of epoxy resin by bacteria was demonstrated by ESEM (Figure 9) and LC-MS (Figure 10). Since the epoxy resin is not soluble in water and maintains its individual droplet shape in solution, ESEM could be used to study the effect of bacteria on the shape and surface morphology of the resin droplet. Two different samples were compared: (1) Davis epoxy resin LY 5052 with a mixture of both bacteria, and (2) Davis epoxy resin LY 5052 without bacteria (a reference sample). Before microscopic observation, the two samples were left in an incubator for one week, after which it was apparent that the sample with bacteria was more turbid. As can be seen in Figure 9, there is a significant difference between the epoxy droplets in both samples. The surface of the droplet in the absence of bacteria (Figure 9a) was quite smooth. In contrast, the droplet surface became rough in the presence of the bacteria mixture (Figure 9b,c). This roughening could be the result of either biofilm formation on the surface of the droplet or surface etching by the bacteria. In both samples, salt residues from the Davis solution were also observed. In Figure 9c, a shape suspected to be a bacterium was analyzed and found to be 1.923 μm long. It should be noted that the shape and size of the two bacteria, as documented by ESEM, match those reported elsewhere [40,41]. 

In order to better understand the metabolic pathway of epoxy resin by the two bacteria, LC-MS analyses were performed. Three sample types were analyzed: (1) Davis epoxy resin LY 5052 with bacteria, (2) Davis epoxy resin LY 5052 without bacteria, and (3) Davis and bacteria without epoxy resin. LC-MS analyses were conducted after one week of incubation. Mass spectrometry analysis of each chromatogram was done both in the positive and in the negative modes (here, only the former is presented, see Figure 10). Examination of the spectra obtained in both MS detection modes revealed distinct differences between the three samples. Peaks with retention times of 1.59 and 1.69 min that appear in the chromatogram of sample (2) do not appear in the chromatograms of samples (1) and (3). These results strongly indicate that the presence of bacteria in the incubation media resulted in consumption of certain constituents. Analysis of the chromatographic peak at 1.59 min and its associated mass spectrum suggested that it most likely indicates the presence of bisphenol A (BPA, molecular weight of 228.3 g/mol) and its dimers and trimer oligomers in the incubation media. BPA is present in sample (2) (retention times between 1.5 and 1.9) as a series of phenolate anion clusters with sodium and potassium cations (in positive mode). Analysis of the mass spectrum that is associated with the chromatographic peak at 1.69 min suggested that this compound is most probably 3,3′-((propane-2,2-diylbis(4,1-phenylene))bis(oxy))bis(propane-1,2-diol) with its 1-(tert-butyl)-4-methylbenzene fragment. Their molecular weights are 376.4 and 148.2 g/mol, respectively.

**Figure 11:**
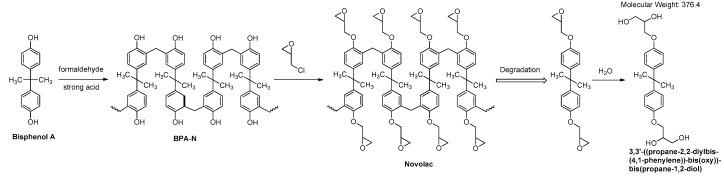
Proposed formation of a novolac resin from bisphenol A and its degradation to 3,3′-((propane-2,2-diylbis(4,1-phenylene))bis(oxy))bis(propane-1,2-diol), which is promoted by bacteria.

This indicates that the bacteria used the epoxy resin as a carbon source. These results are in accordance with the chemical compositions of the two epoxy resins used in this study. Figure 10 suggests that incubation of resin with Davis medium results in some hydrolysis of the soluble resin fraction, yielding BPA as a mixture of phenolate salts, whereas other fragments present in the resin mixture remain intact. The absence of BPA in sample (1), combined with lower concretion of higher molecular weight, is clearly the result of a bacterial metabolism, such as oxidation of the BPA’s methyls, followed by decarboxylation (cleavage of carbon-carbon bond). In Figure 11 we describe schematically the formation of a novolac resin from BPA and its degradation to 3,3′-((propane-2,2-diylbis(4,1-phenylene))bis(oxy))bis(propane-1,2-diol), which is promoted by bacteria. It should be noted that some bacteria capable of biodegrading BPA from soils and the biodegradation process of BPA have already been shown elsewhere [42,43,44]. However, in those publications epoxy resin was not referred to, the bacteria were different, and the biodegradation products were different. With respect to the potential significance of the results of the present study on reduction of environment pollution, it is worth noting that BPA metabolism by organisms has been reported to generally lead to detoxification of BPA [42].

## 4. Conclusions

This article describes our study aimed to isolate from the surroundings microorganisms that can degrade thermoset epoxy resins, which have been considered to be typically non-recyclable and non-biodegradable, in an economically viable process under mild conditions, characterize these microorganisms, demonstrate the biodegradation process, and try understanding the metabolism of epoxy by these microorganisms. Two different epoxy resins were studied: Araldite^®^ LY 5052 and EPON™ resin 815C. Two bacteria were isolated from soil samples collected from areas around an epoxy and polyurethanes manufacturing plant. Based on MALDI-TOF MS, 16S rRNA gene sequencing, VITEK^®^ 2, Gram staining, and epifluorescent microscopy analyses, they were identified as *Rhodococcus rhodochrous* and *Ochrobactrum anthropi*. These bacteria were found to be capable of using epoxy resin as a sole carbon source. The maximal bacterial growth rate was evident at 1 wt.% epoxy resin LY 5052. Bacterial growth took place only when the two bacteria were grown together, indicating an obligatory synergy, which is not yet fully understood. The surface morphology of the epoxy droplets became rough in the presence of the bacteria mixture, due to either biofilm formation on the surface of the droplet or surface etching by the bacteria. The metabolic pathway of epoxy by the two microbes was investigated by LC-MS, which indicated on a possible oxidation of the methyls in Bisphenol A, followed by decarboxylation. The formation of a novolac resin from BPA and its degradation to 3,3′-((propane-2,2-diylbis(4,1-phenylene))bis(oxy))bis(propane-1,2-diol), which is promoted by bacteria, was suggested. This study could pave the way also to biodegradation of the cured end-products of epoxy resins, which might be a more challenging task. Such a microbial degradation process is a promising eco-friendly strategy, which represents a great opportunity to manage waste plastic materials with no adverse impacts.

## References

[1] Acmite Market Intelligence (2017). Global Epoxy Resin Market.

[2] Shah A.A., Hasan F., Hameed A., Ahmed S. (2008). Biological degradation of plastics: A comprehensive review. Biotechnol. Adv..

[3] Pangallo D., Buckova M., Krakova L., Puskarova A., Sakova N., Grivalsky T., Chovanova K., Zemankova M. (2015). Biodeterioration of epoxy resin: A microbial survey through culture-independent and culture-dependent approaches. Environ. Microbiol..

[4] Little B.J., Lee J.S. (2007). Microbiologically Influenced Corrosion.

[5] Wang G., Chai K., Wu J., Liu F. (2016). Effect of *Pseudomonas putida* on the degradation of epoxy resin varnish coating in seawater. Int. Biodeterior. Biodegrad..

[6] Davis B.D., Mingioli E.S. (1950). Mutants of *Escherichia coli* requiring metliionine or vitamin B12. J. Bacteriol..

[7] Janda J.M., Abbott S.L. (2007). 16S rRNA gene sequencing for bacterial identification in the diagnostic laboratory: Pluses, perils, and pitfalls. J. Clin. Microbiol..

[8] Woo P.C.Y., Lau S.K.P., Teng J.L.L., Tse H., Yuen K.-Y. (2008). Then and now: Use of 16S rDNA gene sequencing for bacterial identification and discovery of novel bacteria in clinical microbiology laboratories. Clin. Microbiol. Infect..

[9] Teyssier C., Marchandin H., Jean-Pierre H., Diego I., Darbas H., Jeannot J.L., Gouby A., Jumas-Bilak E. (2005). Molecular and phenotypic features for identification of the opportunistic pathogens *Ochrobactrum* spp.. J. Med. Microbiol..

[10] James G., Schuller M., Sloots T., James G., Halliday C., Carter I. (2010). Universal bacterial identification by PCR and DNA sequencing of 16S rRNA gene. PCR for Clinical Microbiology.

[11] Jeong S.J., Kwon G. (2007). Cloning of fibrinolytic enzyme gene from *Bacillus subtilis* isolated from Cheonggukjung and its expression in protease-deficient *Bacillus subtilis*. J. Microbiol. Biotechnol..

[12] Ligozzi M., Bernini C., Grazia Bonora M., de Fatima M., Zuliani J., Fontana R. (2002). Evaluation of the VITEK 2 system for identification and antimicrobial susceptibility testing of medically relevant Gram-positive cocci. J. Clin. Microbiol..

[13] Deak E., Charlton C.L., Bobenchik A.M., Miller S.A., Pollett S., McHardy I.H., Wu M.T., Garner O.B. (2015). Comparison of the Vitek MS and Bruker Microflex LT MALDI-TOF MS platforms for routine identification of commonly isolated bacteria and yeast in the clinical microbiology laboratory. Diagn. Microbiol. Infect. Dis..

[14] Carbonnelle E., Mesquita C., Bille E., Day N., Dauphin B., Beretti J.L., Ferroni A., Gutmann L., Nassif X. (2011). MALDI-TOF mass spectrometry tools for bacterial identification in clinical microbiology laboratory. Clin. Biochem..

[15] Martiny D., Busson L., Wybo I., El Haj R.A., Dediste A., Vandenberg O. (2012). Comparison of the MICROFLEX LT and VITEK^®^ MS systems for the routine identification of bacteria by matrix-assisted laser desorption-ionization time-of-flight mass spectrometry. J. Clin. Microbiol..

[16] Shapira Y., Multanen V., Whyman G., Bormashenko Y., Chaniel G., Barkay Z., Bormashenko E. (2017). Plasma treatment switches the regime of wetting and floating of pepper seeds. Colloids Surf. B Biointerfaces.

[17] Leahy J.G., Colwell R.R. (1990). Microbial degradation of hydrocarbons in the environment. Microbiol. Rev..

[18] Cheremisinoff N.P. (1996). Biotechnology for Waste and Wastewater Treatment.

[19] Cerny G. (1976). Method for the distinction of Gram-negative from Gram-positive bacteria. Eur. J. Appl. Microbiol..

[20] Chester B., Cooper L.H. (1979). Achromobacter species (CDC group Vd): Morphological and biochemical characterization. J. Clin. Microbiol..

[21] Laffineur K., Janssens M., Charlier J., Avesani V., Wauters G., Delmée M. (2002). Biochemical and susceptibility tests useful for identification of nonfermenting gram-negative rods. J. Clin. Microbiol..

[22] Holmes B., Popoff M., Kiredjian M., Kersters K. (1988). *Ochrobactrum anthropi* gen. nov., sp. nov. from human clinical specimens and previously known as group Vd. Int. J. Syst. Evol. Microbiol..

[23] Hagiya H., Ohnishi K., Maki M., Watanabe N., Murase T. (2013). Clinical characteristics of *Ochrobactrum anthropi* bacteremia. J. Clin. Microbiol..

[24] Chain P.S.G., Lang D.M., Comerci D.J., Malfatti S.A., Vergez L.M., Shin M., Ugalde R.A., Garcia E., Tolmasky M.E. (2011). Genome of *Ochrobactrum anthropi* ATCC 49188T, a versatile opportunistic pathogen and symbiont of several eukaryotic hosts. J. Bateriol..

[25] Torres A.H., Gómez J.R., Vázquez E.G., Gómez J.G. (2014). *Ochrobactrum anthropi* bacteraemia: Report of six cases and review of the literature. Intern. Med..

[26] Mastroianni A., Cancellieri C., Montiní G. (1999). *Ochrobactrurn anthropi* bacteremia: Case report and review of the literature. Clin. Microbiol. Infect..

[27] Alnor D., Frimodt-Meller N., Espersen F., Frederiksen W. (1994). Infections with the unusual human pathogens *Agrobacterium* species and *Ochrobactrum anthropi*. Clin. Infect. Dis..

[28] Chang B.V., Chiang B.W., Yuan S.Y. (2007). Biodegradation of nonylphenol in soil. Chemosphere.

[29] Goodfellow M., Williams S.T., Sharpe M.E., Holt J.G. (1989). Genus *Rhodococcus*. Bergey’s Manual of Systematic Bacteriology.

[30] Bell K.S., Philp J.C., Aw D.W.J., Christofi N. (1998). The genus *Rhodococcus*. J. Appl. Microbiol..

[31] Chen B.S., Otten L.G., Resch V., Muyzer G., Hanefeld U. (2013). Draft genome sequence of *Rhodococcus rhodochrous* strain ATCC 17895. Stand. Genom. Sci..

[32] Larkin M.J., Kulakov L.A., Allen C.C. (2005). Biodegradation and *Rhodococcus*–masters of catabolic versatility. Curr. Opin. Biotechnol..

[33] Martínková L., Uhnáková B., Pátek M., Nešvera J., Křen V. (2009). Biodegradation potential of the genus *Rhodococcus*. Environ. Int..

[34] Larkin M.J., Kulakov L.A., Allen C.C.R., Alvarez H. (2010). Genomes and plasmids in *Rhodococcus*. Biology of Rhodococcus. Microbiology Monographs.

[35] Larkin M.J., De Mot R., Kulakov L.A., Nagy I. (1998). Applied aspects of *Rhodococcus* genetics. Antonie Leeuwenhoek.

[36] Chen X., Schauder S., Potier N., Van Dorsselaer A., Pelczer I., Bassler B.L., Hughson F.M. (2002). Structural identification of a bacterial quorum-sensing signal containing boron. Nature.

[37] Ivanova K., Fernandes M.M., Francesko A., Mendoza E., Guezguez J., Burnet M., Tzanov T. (2015). Quorum-quenching and matrix-degrading enzymes in multilayer coatings synergistically prevent bacterial biofilm formation on urinary catheters. ACS Appl. Mater. Interfaces.

[38] Papenfort K., Bassler B. (2016). Quorum-sensing signal-response systems in Gram-negative bacteria. Nat. Rev. Microbiol..

[39] Li Y.-H., Tian X. (2012). Quorum sensing and bacterial social interactions in biofilms. Sensors.

[40] Li B., Pan D., Zheng J., Cheng Y., Ma X., Huang F., Lin Z. (2008). Microscopic investigations of the Cr(VI) uptake mechanism of living *Ochrobactrum anthropi*. Langmuir.

[41] Li C., Li Y., Cheng X., Feng L., Xi C., Zhang Y. (2013). Immobilization of *Rhodococcus rhodochrous* BX2 (an acetonitrile-degrading bacterium) with biofilm-forming bacteria for wastewater treatment. Bioresour. Technol..

[42] Kanga J.H., Katayama Y., Kondo F. (2006). Biodegradation or metabolism of bisphenol A: From microorganisms to mammals. Toxicology.

[43] Danz E., Sei K., Soda S., Ike M., Fujita M. (2009). Biodegradation of bisphenol A, bisphenol F and bisphenol S in seawater. Int. J. Environ. Res. Public Health.

[44] Eio E.J., Kawai M., Tsuchiya K., Yamamoto S., Toda T. (2014). Biodegradation of bisphenol A by bacterial consortia. Int. Biodeterior. Biodegrad..

